# New year address on the state of psychosomatic medicine in Japan

**DOI:** 10.1186/1751-0759-7-2

**Published:** 2013-01-30

**Authors:** Chiharu Kubo

**Affiliations:** 1 International College of Psychosomatic Medicine, Asian College of Psychosomatic Medicine, Japanese Society of Psychosomatic Medicine, Fukuoka, Japan; 2 Kyushu University Hospital, Fukuoka, Japan

## 

The Japanese Society of Psychosomatic Medicine was founded in 1959, and its first congress was held in 1960, with fewer than 100 members. The society has grown tremendously since then, both in terms of scope and size. In 2012, we had 3,600 members. We have hosted the World Congress of Psychosomatic Medicine two times, first in 1977 in Kyoto, then in 2005 in Kobe, where we were very honored by the attendance of the Emperor and Empress and privileged to hear the Emperor’s encouraging speech.

In 2012, Kyushu University celebrated the 50th anniversary of the founding of the Department of Psychosomatic Medicine, the first such department in Japan. There are currently nine university hospitals with Psychosomatic Medicine departments. Our initial focus was research on the mind/body relationship in typical psychosomatic diseases, such as IBS, bronchial asthma, hypertension, headache, and atopic dermatitis. Great changes have occurred over the past 50 years. With the introduction of new technologies, such as f-MRI and PET scanning, and new knowledge gained by our research, we have been able to shift our focus to the genetic basis of disease and the involvement of environmental factors. This has lead to a change from simple mind/body relationships to a focus on psycho-neuro-immuno-endocrinology.

Psychosomatic medicine has shifted from being on the periphery of medical practice to where it is now considered a core discipline of clinical medicine. We have been able to develop a harmony between evidence based medicine and narrative based medicine. This shift has brought great benefits, but it has also brought about a number of problems that need to be resolved. These include educational, economical, and philosophical issues. In terms of education, we need encourage all universities, not just the current nine, to include extensive teaching of psychosomatic medicine in their curriculums. The Japanese health insurance system must be updated to include provisions that allow the successful treatment of patients with psychosomatic diseases, many of which take more time for treatment than is currently funded. We need to continue our public education efforts to impress on the healthy population the importance of stress management, health promotion, and other forms of preventive medicine.

It is very encouraging that over the past few years we have been able to integrate our efforts to promote psychosomatic medicine with other members of the world community. Our members have been impressively active in their participation in the World Congress of Psychosomatic Medicine and the Asian College of Psychosomatic Medicine, which has grown tremendously in recent years. Our interactions with the world community serve to broaden our perspectives and to promote the perspectives of the Japanese Society, build strong bonds with individual researchers and clinicians, and help us provide the best possible care to our patients.

